# Retinal O-linked N-acetylglucosamine protein modifications: implications for postnatal retinal vascularization and the pathogenesis of diabetic retinopathy

**Published:** 2013-05-21

**Authors:** Zafer Gurel, Kelsey M. Sieg, Keegan D. Shallow, Christine M. Sorenson, Nader Sheibani

**Affiliations:** 1 Department of Ophthalmology and Visual Sciences, University of Wisconsin School of Medicine and Public Health, Madison,WI; 2 Department Pediatrics, University of Wisconsin School of Medicine and Public Health, Madison,WI; 3 McPherson Eye Research Institute, University of Wisconsin School of Medicine and Public Health, Madison,WI

## Abstract

**Purpose:**

Hyperglycemia activates several metabolic pathways, including the hexosamine biosynthetic pathway. Uridine diphosphate N-acetylglucosamine (GlcNAc) is the product of the hexosamine biosynthetic pathway and the substrate for O-linked GlcNAc (O-GlcNAc) modification. This modification affects a wide range of proteins by altering their activity, cellular localization, and/or protein interactions. However, the role O-GlcNAcylation may play in normal postnatal retinal vascular development and in the ocular complications of diabetes, including diabetic retinopathy, requires further investigation.

**Methods:**

The total levels of O-GlcNAc-modified proteins were evaluated by western blot analysis of lysates prepared from retinas obtained at different days during postnatal retinal vascularization and oxygen-induced ischemic retinopathy. Similar experiments were performed with retinal lysate prepared from diabetic Ins2^Akita/+^ mice with different durations of diabetes and retinal vascular cells cultured under various glucose conditions. The localization of O-GlcNAc-modified proteins in the retinal vasculature was confirmed by immunofluorescence staining. The impact of altered O-GlcNAcylation on the migration of retinal vascular cells was determined using scratch wound and transwell migration assays.

**Results:**

We detected an increase in protein O-GlcNAcylation during mouse postnatal retinal vascularization and aging, in part through the regulation of the enzymes that control this modification. The study of the diabetic Ins2^Akita/+^ mouse retina showed an increase in the O-GlcNAc modification of retinal proteins. We also observed an increase in retinal O-GlcNAcylated protein levels during the neovascularization phase of oxygen-induced ischemic retinopathy. Our fluorescence microscopy data confirmed that the alterations in retinal O-GlcNAcylation are similarly represented in the retinal vasculature and in retinal pericytes and endothelial cells. Particularly, the migration of retinal pericytes, but not retinal endothelial cells, was attenuated by increased O-GlcNAc modification.

**Conclusions:**

The O-GlcNAc modification pattern changes during postnatal retinal vascular development and neovascularization, and its dysregulation under hyperglycemia and/or ischemia may contribute to the pathogenesis of the diabetic retinopathy and retinal neovascularization.

## Introduction

The prevalence of diabetes mellitus continues to increase worldwide, with diabetic retinopathy (DR) remaining a leading cause of vision loss in many developed countries. Despite advances in the treatment of both diabetes and DR, the incidence of blindness resulting from DR is rising [1]. The pathogenesis of DR is multifactorial and affects all cell types in the retina. Hyperglycemia-linked pathways, resultant retinal ischemia, and increased vascular permeability augmented by hypertension are common pathways underlying the development and progression of DR [2]. In the late stages of DR, ischemia-induced pathological growth of new blood vessels causes catastrophic loss of vision. Visual loss primarily occurs from proliferative retinal vascularization and/or increased permeability of retinal blood vessels [3]. Although hyperglycemia is recognized as the hallmark symptom of diabetes and its complications, the precise molecular mechanisms affected under hyperglycemic conditions are not well understood.

Hyperglycemia increases O-linked N-acetylglucosamine (O-GlcNAc) modifications in cells, which may play an important role in the pathogenesis of diabetes [4-6]. O-GlcNAc modification is one of the most common posttranslational modifications, and involves a wide-range of proteins including cytoplasmic, mitochondrial, and nuclear ones. Uridine diphosphate (UDP)-GlcNAc, the end product of the hexosamine biosynthetic pathway (HBP), is used for the O-GlcNAc modification of proteins [7]. UDP-GlcNAc is a high-energy molecule that serves as the monosaccharide donor for posttranslational modification by O-GlcNAc transferase (OGT). O-GlcNAcase (OGA) removes O-GlcNAc modification from proteins [7]. This unique and dynamic form of glycosylation occurs by the attachment of O-GlcNAc on the hydroxyl group of serine and/or threonine residues, and it is able to alter the degradation, function, and/or associations of the target protein, similar to phosphorylation.

The regulation of protein function by O-GlcNAc modification is essential during normal developmental processes. Both OGT [8] and OGA [9] knockout mice die early during embryonic development. In addition, dysregulation of O-GlcNAc modification contributes to the etiology of various diseases such as diabetes mellitus [10-13], cardiovascular disease [14-16], cancer [11,17,18], and neurodegenerative disorders. On the other hand, how O-GlcNAcylation contributes to normal developmental processes and/or pathogenesis of various diseases, in particular DR, has yet to be delineated.

The retinal vasculature in mice develops postnatally, and formation of the primary vascular plexus is complete by postnatal day 21 (P21). Organized vascularization is essential not only for the physiological development of the retina, but also for the normal development of most other tissues. Many observations made in the developing mouse retina also apply to developmental angiogenesis in other organs and to tumor vascularization [19]. To our knowledge, no study has examined O-GlcNAc modification in the retina and retinal vascular cells, and its potential role in the pathogenesis of DR remains elusive.

Here, we analyzed total O-GlcNAcylation levels of the retina along with the expression of OGT and OGA, both during postnatal retinal vascular development and in older animals. We also evaluated retinal samples from Ins2^Akita/+^ mice with different durations of diabetes, as well as during oxygen-induced ischemic retinopathy (OIR). Both of these models exhibit unique attributes which allow for the analysis of early and late hyperglycemia-induced retinal vascular changes. The Ins2^Akita/+^ mouse model is useful for studying retinal vascular changes that occur in the early stages of diabetes, while the OIR model is used to analyze the late, proliferative stage of DR [19,20]. We show, for the first time, that total O-GlcNAcylation levels in the retina gradually increase in later stages of maturation as the retinal vasculature develops.

Retinal samples from Ins2^Akita/+^ mice showed higher O-GlcNAcylation levels compared with wild-type mice, especially after 6 weeks of age. In the OIR model, we detected the highest O-GlcNAcylation levels during the neovascularization phase (P15 and P17). We also showed that retinal vascular cells exhibit O-GlcNAcylation of various proteins; their levels increased under high glucose conditions in retinal pericytes (PCs) and endothelial cells (ECs), but not retinal astrocytes (ACs). Increased total O-GlcNAc modification either by high glucose or OGA inhibitors attenuated the migration of retinal PCs but not ECs. Migration of PCs is critical in vascular changes associated with diabetes, including increased permeability and degeneration of the vasculature [21-24]. Together, these findings indicate that O-GlcNAcylation of target proteins is a dynamic process during normal retinal vascularization, and its alterations under both hyperglycemic and ischemic conditions may contribute to retinal vascular dysfunction and neovascularization.

## Methods

### Animals

Wild-type C57BL/6J mice and Ins2^Akita/+^ heterozygous (Akita/+) male mice were obtained from the Jackson Laboratory (Bar Harbor, ME). The Ins2^Akita/+^ colony is maintained by breeding C57BL/6J inbred females with Ins2^Akita/+^ males. Only male Ins2^Akita/+^ mice develop diabetes, and therefore they were used in this research. These mice become diabetic at 4 weeks of age (blood glucose ≥420 mg/dl), and their life expectancy is approximately 10 months (Jackson Laboratory). The majority of early retinopathies in these mice occur after 6 months of diabetes [25]. Control animals were C57BL/6J male littermates. Genomic DNA was prepared from tail biopsies and the transgenic Ins2^Akita/+^ mice were identified by PCR screening (Table 1) The amplified fragments were digested with FNU 4 HI, as recommended by the supplier (New England Biolabs, Ipswich, MA). All procedures were performed according to the Association for Research in Vision and Ophthalmology Statement for the Use of Animals in Ophthalmic and Vision Research and were approved by the Institutional Animal Care and Use Committee of the University of Wisconsin School of Medicine and Public Health.

**Table 1:** List of primers

Name	Forward Primers	Reverse Primers
Ins2^Akita/+^ Screening	5′-TGCTGATGCCCTGGCCTGCT-3′	5′-TGG TCCCACATATGCACATG-3′
OGT	5’-GGCAACCTGGCTTGTGTGT-3’	5’-TGTAGGTATCAATGGCCAGGTCTA-3’
OGA	5’-CAAGTTGCACACAGTGGAGCTAA-3’	5’-AAAGAGGGTGCAGCAACTAAGG-3’
RpL13A	5’-TCTCAAGGTTGTTCGGCTGAA-3’	5’-GCCAGACGCCCCAGGTA-3’

### Mouse model of oxygen-induced ischemic retinopathy

Experiments were performed using wild-type C57BL/6J mice as previously described [20]. One-week-old pups and their mothers were incubated in high oxygen (75%) for 5 days from P7 to P12, and then were returned to room air for 5 days (P17). Mice were sacrificed by CO_2_ inhalation and retinas were collected on postnatal days 7, 12, 15, 17, and 28 for analysis.

### Isolation and culture of primary retinal vascular cells

Retinal vascular cells including retinal ECs, PCs, and ACs were isolated from 4-weeks old C57BL/6-Immorto mice and cultured as we have previously described [26-28]. Multiple isolations of these cells are available in the laboratory and their identity has been confirmed by staining for cell specific markers and analyzed by FACScan caliber flow cytometry.

### Western blot analysis

Retinas were homogenized in cell disruption buffer (mirVana PARIS kit; Invitrogen, Carlsbad, CA). Retinal lysates (30 µg protein) were separated by electrophoresis on precast Tris-Glycin 4%–20% gradient gels (Invitrogen, Carlsbad, CA) and transferred to the Protran nitrocellulose membrane (VWR, Chicago, IL). The membranes were incubated with an anti-O-GlcNAc [RL2] (Abcam, Cambridge, MA), anti-OGT [DM-17] and anti-OGA (Sigma, St. Louis, MO), and anti-β actin antibodies (Thermo Fisher, Chicago, IL). O-GlcNAc competition was performed with 1 M GlcNAc (Sigma 2,423,432). The blots were washed, incubated with appropriate secondary antibody, and developed using enhanced chemiluminescece reagents (ECL; Thermo Fisher). These experiments were repeated at least three times with retinas from three mice.

### RNA purification and quantitative PCR analysis

The total RNA from retina or cells was extracted by mirVana PARIS kit (Invitrogen) according to the manufacturer’s instructions. Cells were allowed to reach 90% confluence, rinsed twice with PBS, scraped from 60 mm tissue culture plates and transferred to Eppendorf tubes. Cells were centrifuged, immediately, frozen in liquid nitrogen, and stored at -80 °C until further analysis. At least 2 retinas were homogenized for RNA purification. The cDNA synthesis was performed with 1 µg of total RNA using Sprint RT Complete-Double PrePrimed kit (Clontech, Mountain View, CA). One microliter of each cDNA (dilution 1:10) was used as a template in quantitative PCR (qPCR) assays, performed in triplicate of three biologic replicates on Mastercycler Realplex (Eppendorf; Hauppauge, NY) using the SYBR qPCR Premix (Clontech) with the specific primers (Table 1). Amplification parameters were as follows: 95 °C for 2 min; 40 cycles of amplification (95 °C for 15 s, 60 °C for 40 s); and a dissociation curve step (95 °C for 15 s, 60 °C for 15 s, 95 °C for 15 s). Standard curves were generated from known quantities for each target gene of linearized plasmid DNA. Ten times dilution series were used for each known targets, which were amplified using SYBR-Green qPCR. The linear regression line for ng of DNA was determined from relative fluorescent units at a threshold fluorescence value (Ct) to quantify gene targets from cell extracts by comparing the relative fluorescent units at the Ct to the standard curve, normalized by the simultaneous amplification of RpL13A (a housekeeping gene) for all samples.

### Immunofluorescence staining for O-linked N-acetylglucosamine modification and retinal vascular plexus

Retinal frozen sections were fixed for 10 min with cold acetone (4 °C), followed by blocking using bovine serum albumin (BSA) PBS-blocking buffer (1% BSA, 0.2% skim milk, 0.3% Triton X-100) for 15 min at room temperature (RT). The sections then were incubated with PBS-blocking buffer containing anti-O-GlcNAc [RL2] (Abcam) and anti-collagen type IV (Millipore, Billerica, MA) for 2 h at RT. Sections were then washed in PBS three times for 5 min each. Cy2- and Cy3-conjugated secondary antibodies (1:600; Jackson ImmunoResearch, West Grove, PA) were applied for 1 h at RT. Sections were washed three times with PBS, covered with PBS/glycerol (2 vol/1 vol), and mounted with a coverslip. Eye sections were viewed and images were captured in digital format using a fluorescence microscope (Axio-Phot, Zeiss, Thornwood, NY).

### Scratch-wound assay

Cells (4×10^5^) were plated in 60 mm tissue culture dishes and allowed to reach confluence (2–3 days). Cell monolayers were wounded with a 1 ml micropipette tip, rinsed twice with Dulbecco's Modified Eagle's Medium (DMEM; Invitrogen) containing 10% fetal bovine serum, and fed with growth medium containing 100 ng/ml of 5-fluorouracil (Sigma) to rule out potential contribution of differences in cell proliferation. The wound closure was monitored and photographed at 0, 24, 48, and 72 h in digital format using a phase microscope. For quantitative assessment, the distances migrated were determined as percentages of total distance using Image J software. These experiments were repeated twice with three different isolations of cells, with similar results.

### Transwell migration assays

Transwell migration assay was conducted as previously described [29]. Briefly, the bottoms of Costar Transwells with an 8 μm pore size (Fisher) were coated with fibronectin (2 μg/ml in PBS) at 4 °C overnight. After being rinsed with PBS, the bottom side of the Transwell was blocked with 2% BSA (Fisher) in PBS for 1 h at room temperature. Retinal ECs and PCs were trypsinized and resuspended in serum-free DMEM, and 1×10^5^ cells in 0.1 ml were added to the top of the Transwell membrane and placed in the wells of a 24-well plate containing 0.5 ml of serum-free DMEM. Cells were incubated for 3 h at 33 °C, fixed with 2% paraformaldehyde for 10 min at room temperature, and stained with hematoxylin and eosin. The stained membranes were mounted on a glass slide, and the number of cells that migrated through the membrane attached to the bottom was determined by counting 10 high-power fields (x200). Transwell migration assays were repeated at least three times for each condition.

### Statistical analysis

Statistical comparisons between specific controls and experimental groups were performed using the one-way analysis of variance followed by a Dunnett post hoc test for significance. For the wound closure assay, a two-way analysis of variance was used followed by a Bonferroni post hoc test for significance. Significant results were denoted by *p<0.05, **p<0.01, ***p<0.001, and ****p<0.0001.

## Results

### Increased O-GlcNAcylation in postnatal developing and aging retinas

**Figure 1:**
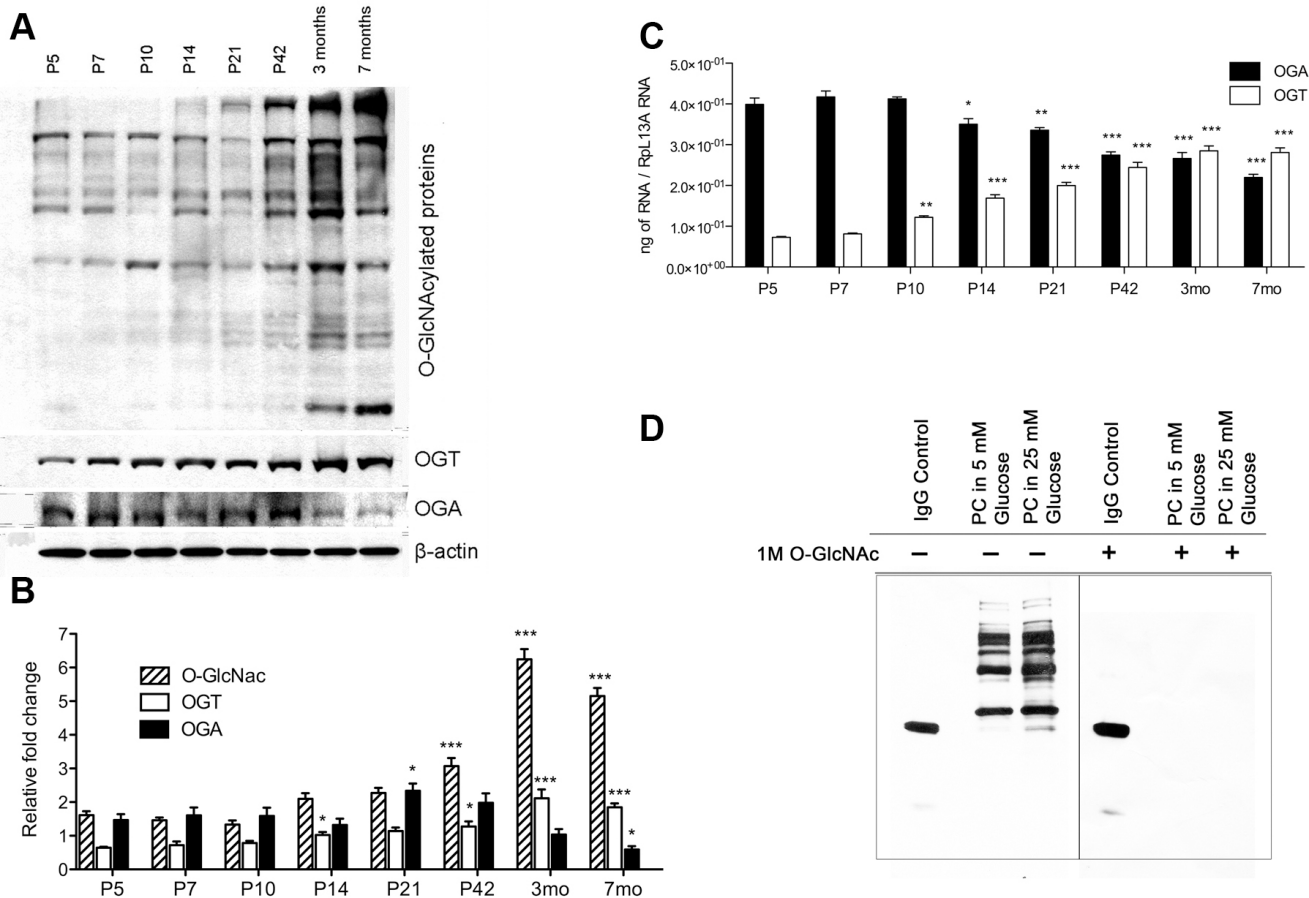
Increased O-linked-N-acetylglucoseamine modification (O-GlcNAcylation) with increased O-GlcNAc transferase (OGT) and decreased O-GlcNAcase (OGA) expression during postnatal retinal vascular development and aging. **A**: Protein lysates (25 µg) from C57BL/6J mouse retinas were analyzed by western blot analysis for O-GlcNAcylated proteins and the expression of OGT and OGA. **B**: The β-actin expression was assessed as a loading control and used for normalization and quantification of data, which were obtained after three different runs (**B**). RNA expression of OGT and OGA were determined by qPCR and normalized by RpL13A RNA expression in samples. The qPCRs were performed with three biologic replicates and in triplicate (**C**). Validation of O-GlcNAc antibody staining of lysates prepared from pericytes (PC) under various glucose conditions. **D**: The GlcNAc (1 M) competition during primary antibody incubation was used to validate the specificity of the O-GlcNAc RL2 antibody. IgG control was used to validate the existence of the secondary antibody. Mean±SEM; * (p≤0.05), ** (p≤0.01), and ***(p≤0.001) significantly different from P5.

To investigate the alterations in O-GlcNAcylated protein levels during postnatal retinal vascularization, we analyzed the levels of O-GlcNAcylation in retinas from P5, P10, P14, P21, and P42 mice. The levels of total O-GlcNAcylated proteins gradually increased during retinal vascularization (Figure 1A,B). In fact, O-GlcNAcylated protein levels continued to increase even after completion of retinal vascularization up to 7 months of age (Figure 1A,B). At the same time, the level of OGT enzyme increased while the level of OGA decreased, as determined by western blotting (Figure 1A,B). A gradual increase in OGT messenger RNA (mRNA) was concurrent with a gradual decrease in OGA mRNA during normal retinal vascularization and later time points (Figure 1C). The OGA mRNA started at a relatively high level at P5 and remained high until P14, when a decrease in the OGA level was observed. In contrast, the OGT mRNA level was relatively low and began to increase by P10. The OGA and OGT mRNAs reached a similar steady-state level by P42; this remained relatively unchanged up to 7 months of age. We did not detect any changes in the expression of glutamine fructose-6-phosphate amidotransferase (GFAT), a rate-limiting enzyme in the HBP, during the times examined (not shown). RL2 antibody was used in western blotting to successfully detect O-GlcNAc-modified proteins [30,31]. Retinal PCs incubated with 25 mM glucose displayed an increased signal for total O-GlcNAc, and coincubation of the antibody with 1 M GlcNAc completely prevented the detection of O-GlcNAc-modified proteins (Figure 1D), validating the specificity of the antibody detection method [32].

Mice are born without a retinal vasculature; retinal vascularization occurs postnatally. A superficial layer of vessels forms during the first week of life by the radial outgrowth of vessels from the optic nerve into the retina, reaching the periphery at approximately P8. By the end of the third postnatal week, all vascular layers are fully formed with multiple interconnecting vessels between layers. These vessels continue to undergo remodeling and pruning and are complete by 6 weeks of age [19]. Interestingly, we showed that the retinal O-GlcNAcylation level starts to increase significantly at 6 weeks of age concomitant with the maturation of the retinal vasculature. Together, these results indicate that O-GlcNAc modification may be important in the development of retinal vasculature and associated with the normal aging process. This elevation of O-GlcNAcylation was programmed and regulated by corresponding gene expression of the OGT and OGA (Figure 1C).

### Increased O-GlcNAcylation in retinas of Ins2^Akita/+^ mice

**Figure 2:**
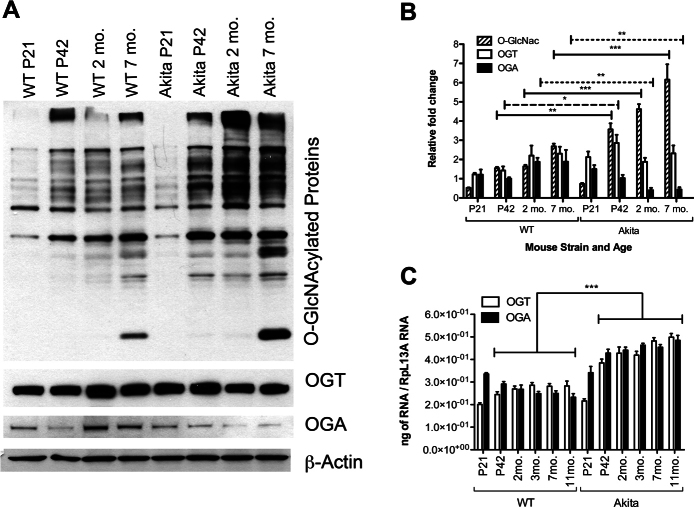
Increased O-GlcNAcylation and decreased O-GlcNAcase (OGA) expression in the retinas of Ins2^Akita/+^ mice. O-GlcNAc transferase (OGT) expression did not show any alterations. **A**: Protein lysates (25 µg) from wild-type C57BL/6J and Ins2^Akita/+^ mice retina were examined by western blot analysis for O-GlcNAcylated proteins and expression of OGT and OGA. **B**: The β-actin expression was assessed as a loading control and used for normalization and quantification of data, which were obtained after three different runs. **C**: Increases in the RNA expression of OGT and OGA were detected in Ins2^Akita^ mice retina by qPCR and normalized to RpL13A RNA expression. The qPCRs were performed with three biologic replicates and in triplicate. Mean±SEM; * (p≤0.05), ** (p≤0.01), and ***(p≤0.001) significantly different from wild-type mice at the same time points of wild-type.

Ins2^Akita/+^ mice carry a mutated insulin2 gene, resulting in improper folding of proinsulin and the loss of pancreatic beta-cells. Ins2^Akita/+^ mice are severely hyperglycemic by 4 weeks of age and develop typical symptoms associated with chronic complications of diabetes, such as retinopathy, neuropathy, and nephropathy [25,33]. The majority of these changes, to various extents, are observed in Ins2^Akita/+^ mice of 6 months of age and older. While there was a modest difference in the levels of O-GlcNAcylation in the retinas from P21 (nondiabetic) Ins2^Akita/+^ mice compared to wild-type mice, a significant increase was observed in Ins2^Akita/+^ retina at all time points after P42 (Figure 2A,B). These differences in the amount of O-GlcNAcylation between Ins2^Akita/+^ and wild-type retinal samples were 2.3-fold at P42, 2.8-fold at 2 months, and 2.3-fold at 7 months. The only significant difference in OGT protein levels between the two groups was observed at P42 (Figure 2B).

Interestingly, the OGA protein level was lower in retinas of 2 and 7 months old Ins2^Akita/+^ mice compared with wild-type mice. However, both OGT and OGA mRNA levels were high in the retinas of Ins2^Akita/+^ mice (Figure 2C). In Ins2^Akita/+^ mice, the OGA and OGT mRNA expression increased slightly over time. Wild-type mice also exhibited increased OGT mRNA over time, while the expression of OGA decreased (Figure 2C). Taken together, these results indicate that hyperglycemia causes a significant increase in O-GlcNAcylated protein levels in the retina of Ins2^Akita/+^ mice. Thus, in addition to increased UDP-GlcNAc under high glucose, decreased OGA enzyme levels may lead to positive feedback in the retina under diabetic conditions.

### Increased O-GlcNAcylation during retinal neovascularization

The mouse model of OIR is a well-established and widely used animal model of retinopathy of prematurity, and mimics the late stages of diabetic (proliferative) retinopathy. The OIR model is characterized by a hyperoxia-induced vessel regression during the first phase and hypoxia-driven proliferative neovascularization during the second phase [19]. Thus, this model allows the investigation of alterations in retinal O-GlcNAcylation levels during retinal neovascularization, which are attributed to switches between vessel regression and angiogenesis. In this model, P7 mice are exposed to 75% oxygen for 5 days to induce impaired development and loss of normal retinal vasculature. Returning the mice to room air at P12 induces an ischemic response and proliferative neovascularization of the retina that peaks at P17. Shortly after, the regression of newly formed vessels begins; by P28, most of the newly formed vessels are regressed [19].

**Figure 3:**
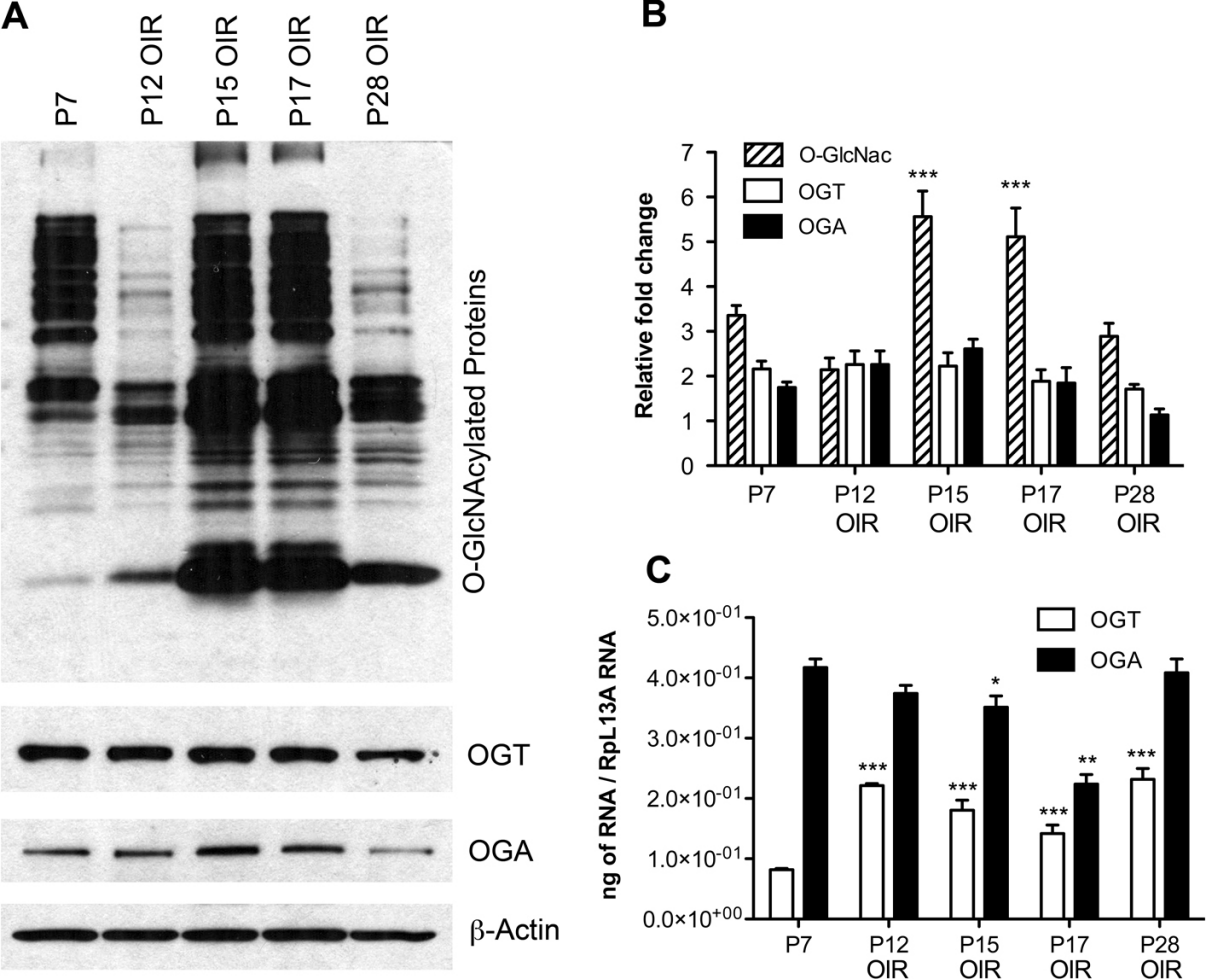
O-GlcNAcylation increases during the neovascularization phase but decreases during the regression phase in an oxygen-induced ischemic retinopathy (OIR) model. O-GlcNAc transferase (OGT) and O-GlcNAcase (OGA) expression did not show any correlation with the alterations in O-GlcNAcylation. **A**: Retinal lysates (25 µg) from C57BL/6J mice during OIR were analyzed by western blot analysis for O-GlcNAcylated proteins and expression of OGT and OGA. **B**: The β-actin expression was assessed as a loading control and used for normalization and quantification of data, which were obtained after three different runs. **C**: RNA expression of OGT and OGA was determined by qPCR and normalized by RpL13A RNA expression in samples. The qPCRs were performed with three biologic replicates and in triplicate. Mean±SEM; * (p≤0.05), ** (p≤0.01), and ***(p≤0.001) significantly different from P7.

We detected increased levels of O-GlcNAc-modified proteins in retinas from P15 and P17 OIR mice during active neovascularization (Figure 3A). Total O-GlcNAcylation was elevated 1.7-fold in P15 and 1.6-fold in P17 retinas compared with P7 retinas (Figure 3B). Relatively lower O-GlcNAcylation was observed during the regression of the existing retinal vasculature in P12 (during exposure to high oxygen) and P28 retinas (during regression of newly formed vessels). The level of O-GlcNAcylated proteins was 1.8-fold lower in P28 retinas compared to P17 retinas. We did not detect any correlation between increased O-GlcNAcylation and OGT or OGA protein or RNA expression during OIR (Figure 3C). These results suggested that other regulatory mechanisms are involved in the alteration of O-GlcNAc modification under hypoxic conditions, such as changes in the activity of OGT and OGA. Thus, O-GlcNAcylation was significantly increased during retinal neovascularization, independent of changes in the expression of OGT and OGA.

### Detection of altered O-GlcNAcylation in the retinal vasculature

**Figure 4:**
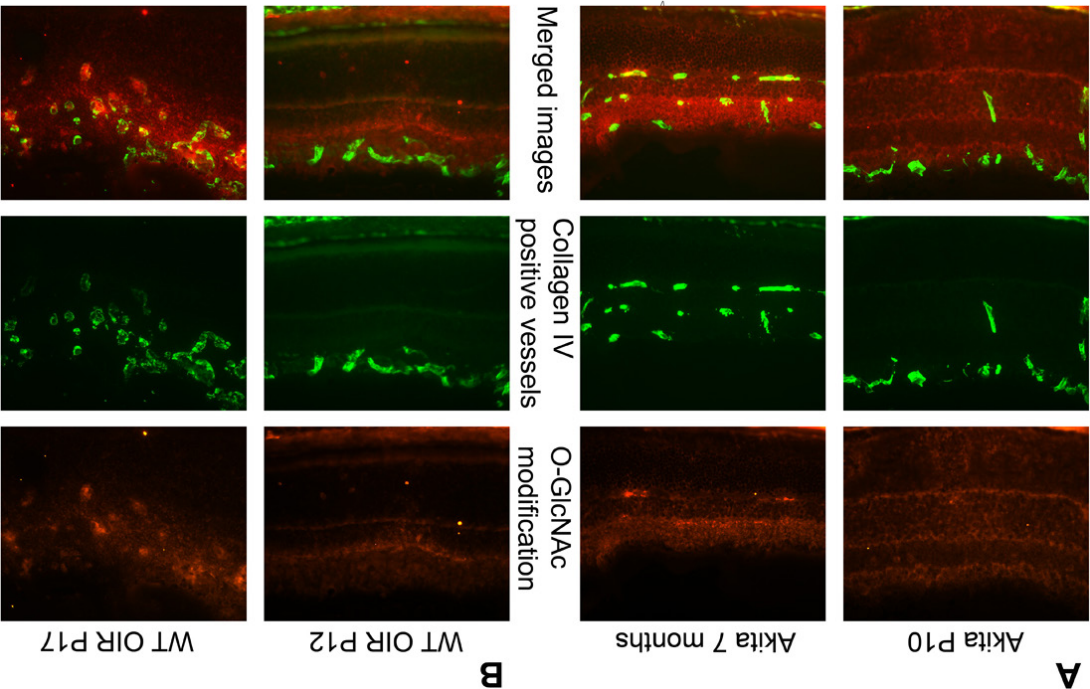
O-GlcNAcylated proteins localize to retinal vascular plexus. **A**: Eye sections from P10 and 7-month-old Ins2^Akita/+^ mice. **B**: P12 and P17 oxygen-induced ischemic retinopathy (OIR) wild-type (WT) mice. O-GlcNAcylated proteins labeled with Cy3 (red, first row), vascular plexus labeled with Cy2 (green, second row) and merge images (third row). Please note the high amount of O-GlcNAcylated protein colocalization with the retinal vascular plexus in 7-month-old Ins2^Akita/+^ and P17 OIR eyes (arrowheads). These images are representative of images evaluated in eyes from at least six mice (original magnification x200).

To combine our retinal results with more direct data from the retinal vasculature, we labeled retinal blood vessels with anti–collagen IV and assessed O-GlcNAc modifications (Figure 4). We detected increased amounts of O-GlcNAcylation in the retinal vessels of adult Ins2^Akita/+^ mice compared to Ins2^Akita/+^ mice younger than 3 weeks old (not diabetic; Figure 4A). Likewise, colocalization of O-GlcNAcylated protein in the collagen IV–positive vessels was detected during retinal neovascularization during OIR (P15 and P17), but this colocalization was not detected when retinal blood vessels were regressing (P12 and P28) in OIR mice (Figure 4B and not shown). These data indicate that increasing amounts of O-GlcNAcylation in the retinal lysates were similarly reflected in O-GlcNAc staining of the retinal vasculature. In this manner, hyperglycemia in Ins2^Akita/+^ mice and neovascularization during OIR caused a significant increase in O-GlcNAc modification of proteins in retinal blood vessels.

### High glucose differentially induced O-linked N-acetylglucosamine modification in retinal vascular cells

**Figure 5:**
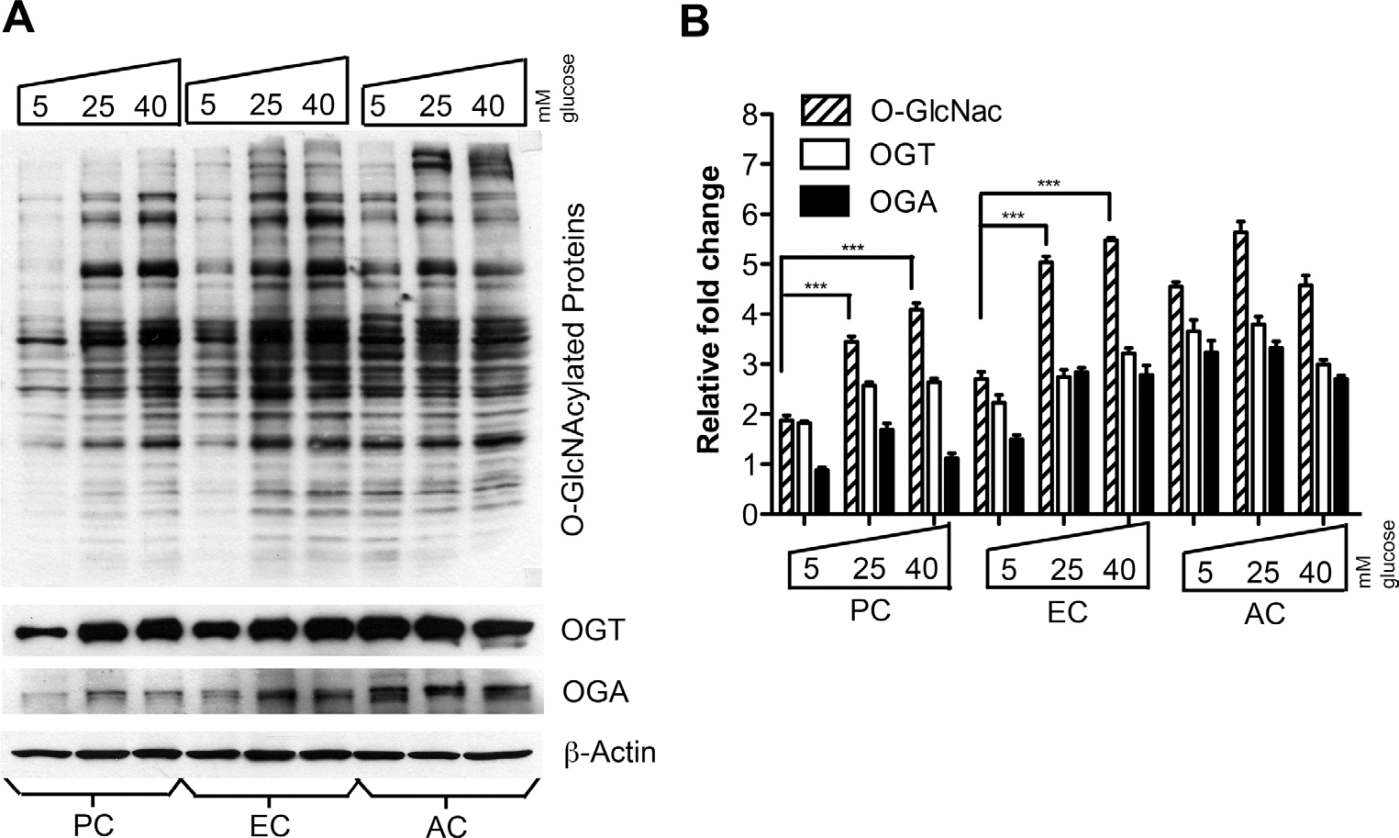
Increased O-GlcNAcylation in retinal pericytes (PC) and endothelial cells (EC), but not astrocytes (AC), under high glucose conditions. **A**: Protein lysates (25 µg) from retinal vascular cells were analyzed by western blot analysis for O-GlcNAcylated proteins and expression of O-GlcNAc transferase (OGT) and O-GlcNAcase (OGA). **B**: The β-actin expression was assessed as a loading control and used for normalization and quantification of data obtained from three different runs (**B**). Mean±SEM; *** (p≤0.001) significantly different from the 5 mM glucose control.

To investigate the effect of high glucose on O-GlcNAc modification in retinal vascular cells, we maintained retinal vascular cells, including ECs, ACs, and PCs, under various glucose concentrations (5 mM, 25 mM, and 40 mM). We detected the lowest O-GlcNAcylation level under low glucose (5 mM) in retinal PCs compared to the other retinal vascular cells. Retinal ECs and PCs responded to high glucose with an approximately twofold increase in the level of protein O-GlcNAcylation compared to their respective low glucose controls (Figure 5). Retinal AC had the highest O-GlcNAcylation levels under low glucose conditions compared to the other retinal vascular cells tested. However, protein O-GlcNAcylation did not increase upon increasing glucose concentration in retinal ACs. The OGT and OGA protein levels increased with high glucose in retinal PCs and ECs (Figure 5). On the other hand, we did not detect a significant change in the mRNA expression for OGT, OGA, or GFAT in retinal vascular cells under various glucose conditions (not shown). These results indicated that the levels of O-GlcNAcylation in different retinal vascular cells varied both at the basal level and under high glucose conditions.

### Attenuation of retinal pericyte migration by increased O-linked N-acetylglucosamine modification

**Figure 6:**
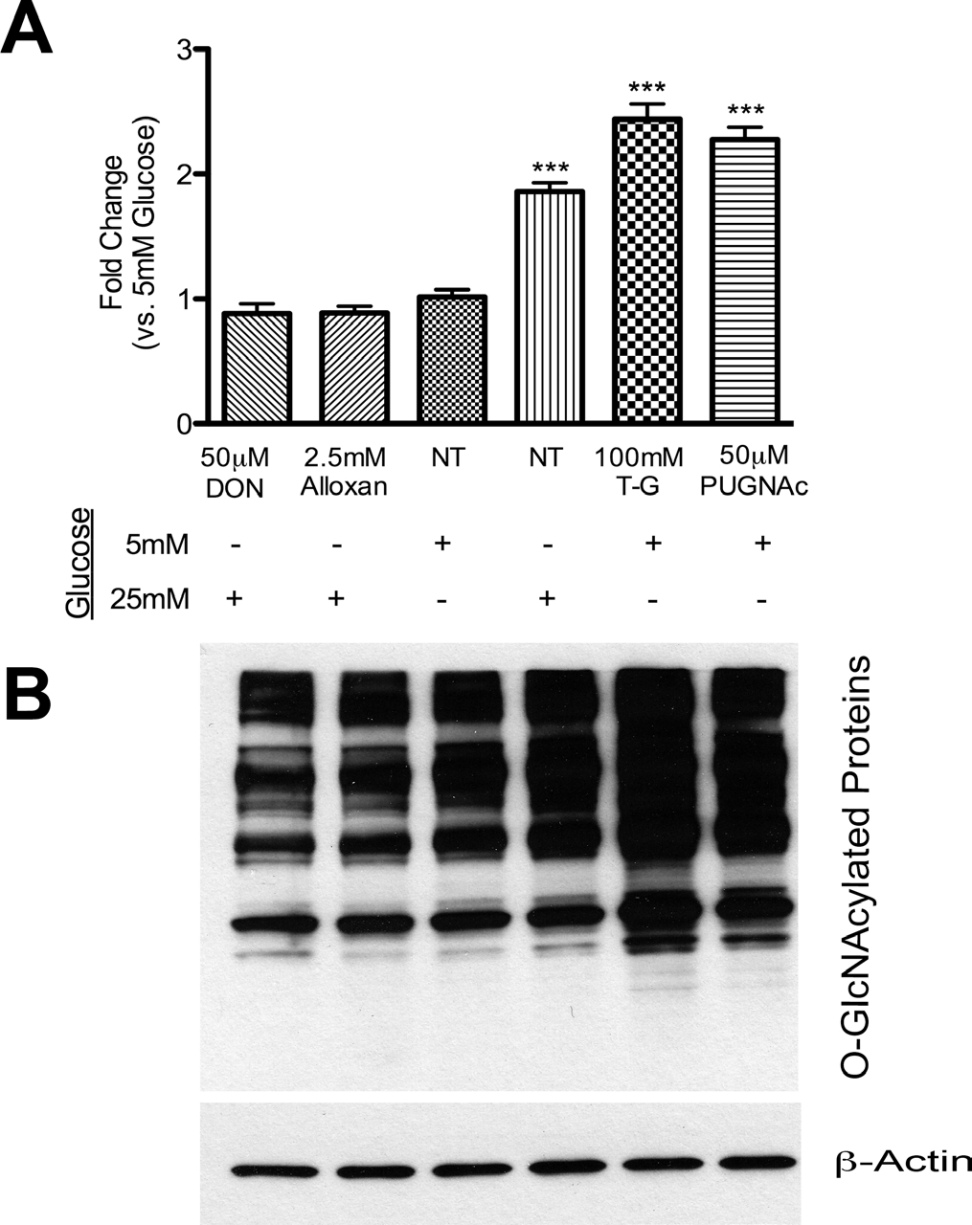
Alterations in the levels of total O-GlcNAc modified proteins in retinal pericytes (PC) by high glucose and specific inhibitors for glutamine fructose-6-phosphate amidotransferase (GFAT), O-GlcNAc transferase (OGT), or O-GlcNAcase (OGA). **A**: Protein lysates (50 µg) from retinal vascular cells were analyzed by western blot analysis for O-GlcNAcylated proteins under 5 mM and 25 mM glucose with or without inhibitors. Thiamet-G and PUGNAc are OGA inhibitors. DON is a GFAT inhibitor and Alloxan is an OGT inhibitor. **B**: The β-actin expression was assessed as a loading control and used for normalization and quantification of data obtained from three different runs. Mean±SEM; *** (p≤0.001) significantly different from 5 mM glucose control.

Migratory features of retinal vascular cells are important to establish healthy capillaries in the retina. We examined the migration of retinal PCs and ECs under various conditions including 5 mM or 25 mM glucose, and the OGA inhibitors Thiamet-G or PUGNAc using a scratch-wound assay. Incubation with high glucose or OGA inhibitors resulted in increased total O-GlcNAc modification in retinal PCs (Figure 6). In contrast, incubation with Alloxan (OGT inhibitor) or DON (GFAT inhibitor) under high glucose conditions decreased total O-GlcNAc modification in retinal PCs.

**Figure 7:**
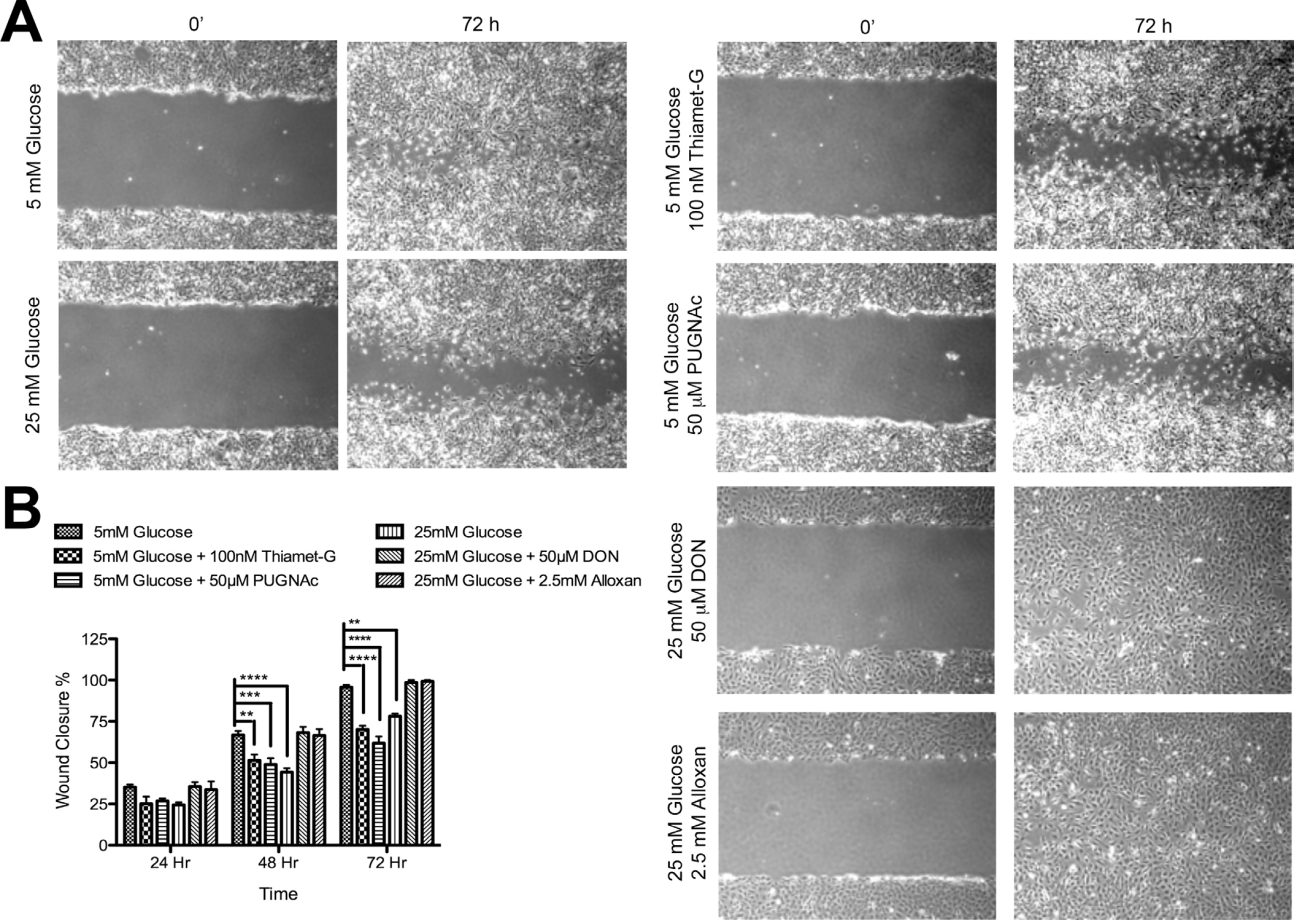
Increased O-GlcNAc modification by high glucose or OGA inhibitors has a negative effect on retinal pericyte (PC) migration. **A**: Cell migration was determined by scratch wounding of PC monolayers, and wound closure at 37 °C was monitored by photography. A representative experiment is shown here. **B**: The quantitative assessment of the wound closure (**p<0.01, ***p<0.001, and ****p<0.0001; n=3).

**Figure 8:**
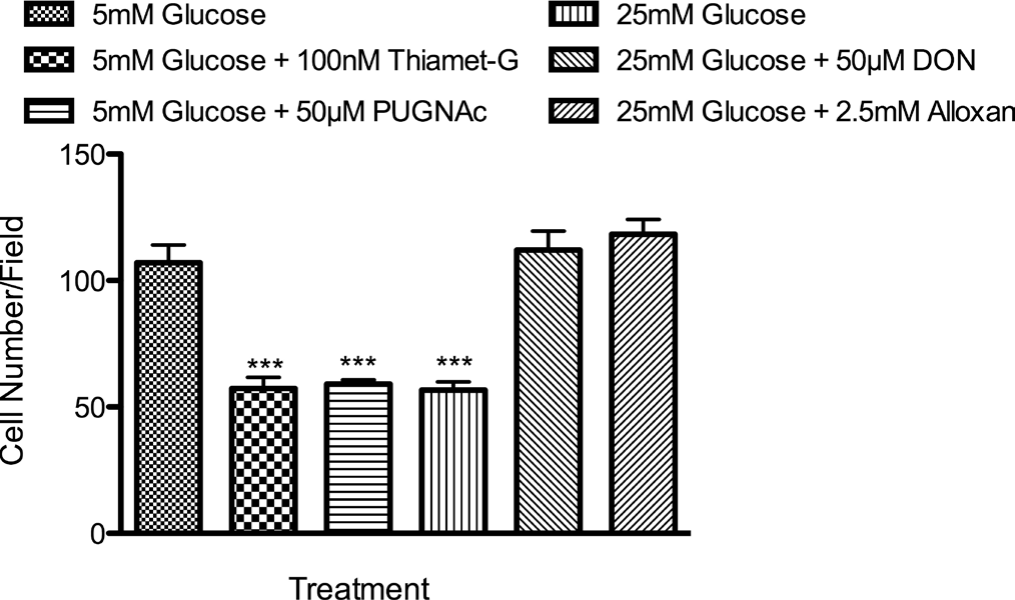
Transwell migration assay confirmed the migration results observed in the scratch-wound assay. Transwell assays were performed at 33 °C with retinal PC cultured under various conditions. Cells that migrated through the membrane were compared with their control group grown in 5 mM glucose (***p<0.001; n=3).

To assess the impact of these treatments on cell migration, a confluent monolayer of retinal PCs, cultured under various glucose conditions for 5 days, was wounded and returned to 37 °C in the presence of 5-fluorouracil (100 ng/ml) to prevent cell proliferation. The wound closure was monitored daily using a phase microscope equipped with a digital camera. Retinal PCs cultured in the presence of 25 mM glucose or OGA inhibitors migrated significantly more slowly than PCs cultured in 5 mM glucose (Figure 7). Similar results were observed using a transwell migration assay (Figure 8). Incubation with OGA inhibitors or 25 mM glucose caused a twofold decrease in the number of PCs migrating through the membrane compared with their control group grown in 5 mM glucose. In contrast, the migration of retinal ECs under various glucose conditions or OGA inhibitors was not affected (not shown).

We next determined whether the OGT inhibitor (Alloxan) or the GFAT inhibitor (DON) was able to prevent the negative effects of high glucose on retinal PC migration. We observed that Alloxan and DON were able to decrease total O-GlcNAc modification under high glucose conditions to normal glucose levels (Figure 6), and restored the migration of retinal PCs to that observed in normal glucose levels (Figure 6, Figure 7, and Figure 8). Thus, we concluded that increased O-GlcNAc modification negatively impacts the migration of retinal PCs, but not retinal ECs. Together, our data indicate that a high glucose–mediated increase in O-GlcNAc modifications impairs retinal PC migration.

## Discussion

Here, we demonstrated that total protein O-GlcNAcylation was increased during postnatal retinal vascularization and with aging. The total protein O-GlcNAc modification was highest in the retinas from diabetic and OIR mice during active neovascularization. We also showed that the increased retinal O-GlcNAc modification correlated with the upregulation of O-GlcNAc modifications in the retinal vasculature and retinal vascular cells. In addition, the migration of retinal PCs was attenuated with increased total O-GlcNAc modification in the presence of glucose or OGA inhibitors, and restored in the presence of OGT inhibitors under high glucose conditions. To our knowledge, these are the first report of alterations in retinal O-GlcNAc modifications during postnatal retinal vascularization, diabetes, and OIR, as well as retinal vascular cells under various glucose conditions. The identity of the specific proteins targeted by O-GlcNAc modification in retinal vascular cells, as well as their role in the regulation of retinal vascular function, will be a subject of future investigation in our laboratory.

O-GlcNAc modification is one of the most common but least studied posttranslational modifications. It is thought to be important in the pathogenesis of diabetes, since hyperglycemia activates both the HBP pathway and O-GlcNAc modification. Interestingly, we detected relatively low protein O-GlcNAcylation during the first three weeks of postnatal life, and after 4 weeks of age, a gradual increase in retinal protein O-GlcNAc modification was observed. Our finding of lower retinal O-GlcNAcylation during postnatal retinal vascularization is consistent with the findings on the antiangiogenic effects of O-GlcNAcylation [34]. However, a gradual increase in the level of protein O-GlcNAcylation was detected in retinas from older mice. These results were consistent with a recent study reporting increased O-GlcNAcylation levels in various tissues including the brain, lung, skin, thymus, testis, and liver of older mice compared with younger mice [9]. Thus, O-GlcNAc modification may be important in retinal vascular homeostasis and increase with the aging process. Furthermore, this elevation of O-GlcNAc modification in the retina was programmed and controlled by expression of genes responsible—OGT and OGA—in the regulation of this modification. These results may also explain the high incidence of dysregulated O-GlcNAc modification, especially in diseases more common in older adults, including diabetes mellitus [10,12,13], cardiovascular disease [14-16], cancer [11,17,18], and Alzheimer disease [35-37]. However, the exact contribution of these modifications to the pathogenesis of these diseases remains to be investigated.

Ins2^Akita/+^ mice spontaneously develop diabetes with a rapid onset [25,33] and represent an excellent model for exploring the molecular mechanisms involved in the initiation and early progression of diabetes and its complications, including DR [25]. We found elevated O-GlcNAc modifications at 6 weeks of age (P42), as well as decreased OGA in the retina of male Ins2^Akita/+^ mice compared to wild-type mice. Interestingly, the RNA levels of OGA and OGT did not correlate with protein levels in these mice. The RNA expressions of both OGT and OGA were relatively high in the retina of Ins2^Akita/+^ mice. During hyperglycemia with activation of the HBP, OGT and OGA expression may be upregulated to manage the increase in the level of UDP-GlcNAc. However, posttranslational mechanisms are likely to be involved in the regulation of OGT and OGA under hyperglycemic conditions. These posttranslational mechanisms may be in part due to the short half-life of these enzymes, especially OGA in the retina. Notably, we showed elevated levels of O-GlcNAcylation in Ins2^Akita/+^ mice retina, further implicating O-GlcNAcylation involvement in the pathogenesis of DR. However, additional studies are required to establishing a direct role of increased O-GlcNAc modification and the pathogenesis of DR.

While the Ins2^Akita/+^ mouse is a useful model to investigate the early stages of diabetes, the OIR model is commonly used to study late (proliferative) stages of DR in mice. Using the OIR model, returning mice to room air after hyperoxia at P12 triggers retinal neovascularization, which at P17. Likewise, in the late stages of DR, the ischemia-driven pathologic growth of new blood vessels causes a catastrophic loss of vision. We observed a significant increase in the levels of total O-GlcNAcylation in the retinas of mice at P15 and P17 during OIR. At first glance, these data appeared to contradict our findings during normal postnatal retinal vascularization, where we detected lower O-GlcNAcylation levels during normal angiogenesis (Figure 1). This contrast, however, can be explained by the involvement of different mechanisms in these processes, which will be the subject of future investigation.

It may be preemptive to suggest that the function of O-GlcNAcylation modifications during angiogenesis is universally understood as antiangiogenic. Under normal conditions, O-GlcNAc modifications act as an angiogenesis blocker, as our data on normal postnatal retinal vascularization suggested; moreover as demonstrated by Luo et al., O-GlcNAcylation modifications have an antiangiogenic role in the vasculature of animal models of both type 1 and 2 diabetes [34]. However, this is inconsistent with data depicting increased angiogenesis in proliferative DR, despite the existence of high O-GlcNAc protein modification [38]. There are circumstances where O-GlcNAcylation may be involved in proangiogenic pathways, including during OIR (this study) and tumor progression [18,39,40]. Although no O-GlcNAcylated protein was detectable in retinal vessels by fluorescence microscopy in young wild-type or Ins2^Akita/+^ mice, high levels of O-GlcNAcylation-positive cells were detected in the retinal vasculature of 3 month and older Ins2^Akita/+^ mice, and active neovascularization stages during OIR were observed. Thus, alterations in total O-GlcNAcylation and its regulating enzymes in total retinal extract were associated with a similar trend observed in the retinal vasculature. Additional studies are needed to further delineate the role of O-GlcNAc protein modification in the regulation of angiogenesis in various tissues and pathological conditions.

We showed that the level of O-GlcNAcylation was also altered in different retinal vascular cells under various glucose conditions. Retinal PCs and ECs responded to high glucose through a significant increase in O-GlcNAc modification. However, retinal AC did not show a significant alteration in O-GlcNAcylation levels under high glucose conditions. Pericytes are known to be the cell type that is primarily and most affected by hyperglycemia during the early stages of diabetes [41]. Interestingly, retinal PCs had the lowest level of O-GlcNAcylation compared to the other retinal vascular cells under the low glucose condition. These results suggest that the increasing level of O-GlcNAc modification may be a determining factor in the sensitivity of retinal vascular cells with low basal levels of O-GlcNAcylation, such as PCs, to hyperglycemia. These observations are consistent with increased total O-GlcNAc modification negatively affecting retinal PC, but not retinal EC, migration. The formation of a substantial and functional retinal vascular unit requires interactions between PCs and ECs. In this manner, our data regarding the negative impact of increased O-GlcNAc modification under high glucose on retinal PC migration, may explain altered permeability and leaky abnormal new vessels in DR.

To our knowledge, a potential role for O-GlcNAc modification in abnormal angiogenesis during DR and OIR, as well as in retinal vascular cells, has not been previously demonstrated. This study provides strong evidence regarding the possible contribution of dysregulated O-GlcNAc modification to the pathogenesis of DR and retinal neovascularization. Understanding the molecular mechanisms by which retinal vasculature is dysregulated during diabetes will aid in the development of new therapeutic strategies. Thus, interrupting disease progression via regulation of O-GlcNAcylation modifications may provide an alternative target for the treatment of DR.
